# The clinical characteristics of autistic women with restrictive eating disorders

**DOI:** 10.1192/bjo.2024.65

**Published:** 2024-07-26

**Authors:** Janina Brede, Charli Babb, Catherine R.G. Jones, Lucy Serpell, Laura Hull, James Adamson, Hannah Baker, John R.E. Fox, Will Mandy

**Affiliations:** Research Department of Clinical, Educational and Health Psychology, University College London, UK; School of Psychology, Cardiff University, UK; ; and Eating Disorder Service, North East London NHS Foundation Trust, London, UK; Centre for Academic Mental Health, Population Health Sciences, Bristol Medical School, University of Bristol, UK; Doctorate in Clinical Psychology, Primary Care and Mental Health, University of Liverpool, UK

**Keywords:** Autistic spectrum disorders, feeding or eating disorders, anorexia nervosa, mental health services, neurodevelopmental disorders

## Abstract

**Background:**

Autistic women are at high risk of developing restrictive eating disorders (REDs), such as anorexia nervosa.

**Aims:**

This study provides an overview of the clinical characteristics of autistic women with REDs to (i) enhance understanding of increased risk, and (ii) support the identification of autistic women in eating disorder services.

**Method:**

We compared self-reported autistic and disordered eating characteristics of: autistic participants with REDs (Autism + REDs; *n* = 57); autistic participants without REDs (Autism; *n* = 69); and women with REDs who are not autistic (REDs; *n* = 80). We also included a group of women with high autistic traits (HATs) and REDs, but no formal autism diagnosis (HATs + REDs; *n* = 38).

**Results:**

Autism + REDs participants scored similarly to Autism participants in terms of autistic characteristics and to REDs participants in terms of experiencing traditional disordered eating symptoms. Autism + REDs participants were distinguished from both groups by having more restricted and repetitive behaviours and autism-specific eating behaviours related to sensory processing, flexibility and social differences. HATs + REDs participants showed a similar pattern of scores to Autism + REDs participants, and both also presented with high levels of co-occurring mental health difficulties, particularly social anxiety.

**Conclusion:**

The presentation of autistic women with REDs is complex, including both traditional disordered eating symptoms and autism-related needs, as well as high levels of co-occurring mental health difficulties. In eating disorder services, the REDs presentation of autistic women and those with HATs should be formulated with reference to autism-specific eating behaviours and co-occurring difficulties. Treatment adaptations should be offered to accommodate autistic characteristics and related needs.

Autism spectrum disorder (henceforth ‘autism’) is a lifelong neurodevelopmental condition characterised by differences in social relating, communication, flexibility and sensory processing. Currently, autistic individuals, compared to non-autistic individuals, have a high risk of developing mental health problems, but a low chance of receiving effective help for these difficulties.^1^ In part, this is because the current evidence base to guide formulation, prevention and treatment of mental health problems for autistic individuals is limited.^2^

In recent years it has become clear that autistic girls and women are at increased risk of developing restrictive eating disorders (REDs), defined here as encompassing anorexia nervosa (i.e. restrictive eating behaviours and/or compensatory behaviours resulting in significantly low body weight, driven by weight and shape concerns), atypical anorexia nervosa (i.e. meeting criteria for anorexia nervosa, but without being substantially underweight) and avoidant/restrictive food intake disorder (ARFID) (i.e. clinically significant restrictive eating that is not driven by weight and shape concerns). Around 20% of women with an anorexia nervosa diagnosis meet criteria for autism;^3^ and rates of ARFID are also elevated in autistic individuals.^4^ REDs have serious consequences for physical and mental health, functioning, quality of life and mortality.^5^

Although there is a developing research literature on the overlap between autism and REDs,^6^ thus far, we lack a systematic, empirical description of the clinical characteristics of autistic women with REDs using validated measures of both autistic and eating disorder features. This gap in knowledge constrains quality of care for two reasons. First, it limits our understanding of why autistic women are at elevated risk for REDs, which hinders progress towards developing effective interventions. Autistic women, and those with high autistic traits, benefit less from current eating disorder interventions and care pathways than women with low autistic traits.^7^ They have lower recovery rates and longer illness duration, and require more intense treatment.^8–10^ Further, autistic women tend to report that their autism is generally not accommodated in eating disorder services, such that their needs are not met, and they find conventional eating disorder treatments hard to access and/or ineffective.^11,12^ This reduced effectiveness of care for autistic women with REDs may partially reflect the fact that their REDs have autism-related causal and maintaining factors, which are not addressed by treatment models which were developed with and for non-autistic people.^13^ Instead, treatments often target ‘traditional’ symptoms, such as underlying weight and shape concerns,^14^ which may be less prominent in autistic individuals with REDs.^13^

Second, the current lack of an account of the clinical presentation of autistic women with REDs limits attempts to identify autistic women in eating disorder services. Usually, autistic women with a RED present to services without an autism diagnosis, and those who are subsequently identified as autistic tend to experience long (~8 years) delays before their diagnosis.^11^ When their autism is unrecognised, it impedes their ability to access and benefit from appropriate clinical care.^12^ Identifying autistic women with REDs is difficult, because (i) it is challenging to assess autism in adult women,^15^ and (ii) behaviours superficially presenting as autistic traits (e.g. rigidity, differences in social cognition) can arise as psychological effects of starvation and thus may be misinterpreted by clinicians.^16^ A description of the clinical autistic characteristics and disordered eating symptoms of autistic women with REDs can help improve their identification in eating disorder services.

## Aims

We aim to describe the autistic characteristics (autistic traits in adulthood and childhood, restricted and repetitive behaviours, camouflaging behaviours) and disordered eating symptoms (traditional eating disorder symptoms, body image issues, pride in eating pathology, autism-specific unusual eating behaviours) of autistic individuals with REDs (Autism + REDs), compared with autistic individuals without REDs (Autism) and women with REDs who are not autistic (REDs).

A subsidiary aim is to describe a group of women with high autistic traits and REDs, but no formal autism diagnosis (HATs + REDs), to better understand their presentation and thereby help services to identify clients who might benefit from autism assessments and treatment modifications. In addition, we test whether autistic traits were correlated with body mass index (BMI) in each group to assess whether those with more autistic traits weighed less, which would support the theory that autistic traits in RED populations are in part driven by the effects of starvation.

## Ethics statement

Written informed consent was obtained from all participants in this study. Full ethical approval was gained from the UCL Research Ethics Committee (12973/002), the Health Research Authority and Health and Care Research Wales (19/WA/0303).

## Method

### Procedure

Two autistic women with experience of REDs reviewed the study protocol and materials and advised on how to make the study accessible for potential participants.

Recruitment was via UK National Health Service (NHS) eating disorder and autism services, social media and charities, with a £15 participation incentive. After eligibility screening and written informed consent, participants provided data via an online survey. Data collection started prior to COVID-19, and the protocol was adapted so that the study could continue during the pandemic; please see Supplementary Appendix 1 available at https://doi.org/10.1192/bjo.2024.65 for information on measures taken to minimise the pandemic's impact upon data collection. Data were collected as part of a larger project. Some of the data presented in the current study have also been included in two PhD theses^17,18^ and a publication on autistic women's eating disorder service experience.^12^

### Measures

#### Background measures

BMI was calculated based on participants’ self-reported current height and weight. Conventionally, those with a BMI below 18.5 are categorised as ‘underweight’, between 18.5 and 24.9 as ‘healthy’ and over 25 as ‘overweight’.

Depression and anxiety were measured with the Hospital Anxiety and Depression Scale (HADS^19^), a self-report questionnaire. The maximum possible score on each subscale (anxiety/depression) is 21. Scores of 0–7 are considered indicative of non-clinical levels of anxiety and depression, of 8–10 indicative of borderline, and of 11 indicative of high levels of anxiety or depression. Internal consistency in the current sample for HADS anxiety (*α* = 0.81) and depression (*α* = 0.83) subscales was high.

Social anxiety was measured using the Social Phobia Inventory (SPIN^20^). Spin total scores can range from 0 to 68. Internal consistency in the current sample was excellent (*α* = 0.91).

#### Autistic characteristics

The Ritvo Autism Asperger Diagnostic Scale-14 (RAADS-14^21^) is an adult autism screening measure. RAADS-14 total scores were used in the current study to split participants with REDs without an autism diagnosis into the REDs group and HATs + REDs group to reduce the chance that the REDs group (which we intended to be without autistic participants) included undiagnosed autistic women. In addition to enhancing the internal validity of our groups, this allowed us to explore the presentation of those with an eating disorder, no autism diagnosis and high autistic traits. RAADS-14 total scores range between 0 and 42. For allocation to the HATs + REDs group, we used the threshold score of 23, which is recommended for psychiatric populations^21^ and has been used in previous studies to identify individuals unlikely to meet criteria for an autism diagnosis.^22^ In addition, we used the RAADS-14 to calculate the RAADS-14 childhood ratio. For each RAADS-14 item, participants recorded whether it applied only when they were a child, only now, neither or both. The RAADS-14 childhood ratio, ranging from 0 to 1, considers the number of items endorsed to have been present in childhood relative to the total number of items endorsed: higher scores indicate the presence of more autistic characteristics since childhood. Thus, a higher score supports the presence of ‘true autism’, as opposed to a phenocopy arising after the onset of an eating disorder.

The Adult Autism Spectrum Quotient^23^ is a 50-item measure of autistic-like traits and behaviours in the general population and has been widely used, including with individuals with eating disorders.^24^ Total adult autism spectrum quotient scores can range from 0 to 50, with a recommended screening threshold score of 26 in clinical populations.^25^ Only participants who participated after the onset of COIVD-19 completed this measure (see Supplementary Appendix 1). Internal consistency in the current sample was high (*α* = 0.89).

The Adult Repetitive Behaviours Questionnaire (RBQ-2A^26^) measured autism-related restricted and repetitive behaviours. Possible total RBQ-2A scores range from 20 to 60. Internal consistency in the current sample was excellent (*α* = 0.91).

The Camouflaging Autistic Traits Questionnaire (CAT-Q^27^) measured autism-related camouflaging behaviours, with possible CAT-Q total scores ranging from 25 to 175. Internal consistency in the current sample was excellent (*α* = 0.93).

#### Disordered eating symptoms

The Eating Disorder Examination-Questionnaire (EDE-Q^28^) was used to assess eating disorder symptoms across four subscales: dietary restraint, eating concern, weight concern and shape concerns. The current paper reports the global score across subscales, referred to as ‘traditional eating disorder symptoms’. Mean EDE-Q global scores can range from 0 to 6, with a threshold score of 2.5 being recommended for screening purposes.^29^ Internal consistency was excellent (*α* = 0.96) in the current sample.

The Body Shape Questionnaire (BSQ^30^) measured preoccupations with body image and concerns about body shape, with possible BSQ total scores ranging from 34 to 204. Scores of less than 81 suggest little or no worry about body shape, whereas scores of more than 140 suggest extreme worry about body shape, with a total possible score range.^30^ There was a high internal consistency in the current sample (*α* = 0.89).

The Pride in Eating Pathology Scale (PEP-S^31^) measured pride towards eating disorder symptoms such as food restriction and weight loss. The PEP-S total score can range from 49 to 343. Internal consistency was excellent (*α* = 0.98).

The SWedish Eating Assessment for Autism Spectrum Disorders (SWEAA^32^) measured unusual eating behaviours and eating disturbances common for autistic adults. The measure is divided into eight subscales, each including 2–11 items (‘perception’, ‘motor control’, ‘purchase of food’, ‘eating behaviour’, ‘mealtime surroundings’, ‘social situation at mealtimes’, ‘other behaviours associated with disturbed eating’, ‘hunger/satiety’), and two single items (‘pica’, ‘simultaneous capacity’). A brief description of each subscale is provided in Supplementary Appendix 2, sTable 1. Each subscale score is transformed into a scale from 0 to 100 to aid interpretability. Internal consistency of subscales was high or acceptable (all *α* ≥ 0.68), apart from for the SWEAA hunger/satiety subscale (*α* = 0.32), which may reflect its small number (*n* = 2) of items.

### Participants

There was insufficient existing literature from which to estimate anticipated group difference effect sizes to inform sample size/power calculations. Thus, we chose, a priori, to power this study to be sensitive to detect difference of an effect size estimated to be of clinical importance (medium-large effect size; Cohen's d ≥ 0.6 with two-tailed alpha at 0.05) and identified a minimum target sample size on this basis. Participants were recruited in three distinct groups: (i) those who have an autism diagnosis and do not have REDs (Autism group); (ii) those who have a diagnosis of autism and a RED (Autism + REDs group); and (iii) those with a RED diagnosis, without an autism diagnosis. Autism and eating disorder diagnoses were self-reported. Steps taken to verify diagnostic status and other inclusion criteria are outlined in Supplementary Appendix 3. Prior to analysis, participants with REDs without an autism diagnosis were then split into the REDs group and HATs + REDs group. The final sample comprised 244 participants meeting inclusion criteria (Autism *n* = 69; Autism + REDs *n* = 57; HATs + REDs *n* = 38; REDs *n* = 80). A post-hoc sensitivity analysis determined that all group comparisons were powered to detect medium-large effects (i.e. Cohen's d ≥ 0.6) (Supplementary Appendix 4, sTable 3).

### Analysis

The raw data were inspected for missing responses. Levels of missing were generally low for both scale- and item-level data. For none of the questionnaires were more than 2.4% of the data missing, BMI and adult autism spectrum quotient excepted. For BMI there were likely reasons related to their eating disorder why participants did not report their height or weight. For the adult autism spectrum quotient scores there were methodological reasons for missing data (see Supplementary Appendix 1). Non-available data for these variables were treated as missing and pairwise deletion was employed. Little's Missing Complete at Random (MCAR) test was carried out on all other variables. The tests were non-significant for all measures, indicating no pattern to missing data. Given that data were likely to be missing completely at random and less than 5% were missing, we opted to impute missing data to retain statistical power. We followed the Treatment and Reporting of Missing Data in Observational Studies framework,^33^ using multiple imputation and pre-specified sensitivity analyses to compare imputed and complete case analyses. Item-level missing data were addressed using multiple imputation by chained equations in R, drawing on all available variables as statistical predictors. We imputed five data-sets, combining estimates using Rubin's rules.^34^ Winsorising was used to substitute outliers. To identify outliers, each variable, split by group, was assessed using the outlier-labelling rule, which proposes an interquartile range multiplier approach to detect outliers.^35^ The current study employed a multiplier of 2.2, which is considered most sensitive.^35^ Results are based on the imputed data-set, with any differences in the senstivity analysis being reported.

Spearman's rank-order correlations between autistic traits (RAADS-14 total) and BMI were calculated for each group to assess the relationship between autistic traits and low weight, which was used as an indicator of starvation.

Groups were compared on each dependent variable using one-way analysis of covariances (ANCOVAs), adjusting for group differences in age. Non-parametric alternatives were used where assumptions had been violated. To assess group differences in the pattern of subscale scores on the SWEAA, we conducted mixed-design ANCOVAs on nine of the ten subscales. The SWEAA pica subscale was not included as its data varied widely from a normal distribution.

## Results

### Descriptive

Demographics and clinical background variables for each group are presented in Table 1. Participants in the Autism group were significantly older than participants in the other three groups and were on average diagnosed as autistic later than Autism + REDs participants. On the HADS, the Autism group scored significantly lower for both depression and anxiety than the other three groups, who all scored similarly. Regarding social anxiety, the Autism + REDs and HATs + REDs groups both scored significantly higher than the other two groups.

**Table 1:** Means (s.d.) and frequencies (%) for demographic and clinical background variables for each group

		Autism (*n* = 69)	Autism + REDs (*n* = 57)	REDs (*n* = 80)	HATs + REDs (*n* = 38)	Main effect^a^	Group comparison and significant post-hoc comparisons (Bonferroni correction applied for multiple comparisons with adjusted significance level at *P* ≤ 0.0083)
Gender	Female	64 (92.8%)	50 (87.7%)	80 (100%)	38 (100%)	Fisher–Freeman–Halton Exact Test (2-sided): *P* = 0.001	
	Agender/gender neutral/non-binary/gender-fluid/other/prefer not to say	5 (7.2%)	7 (12.3%)	0	0		
Age^b^	Current age in years mean (s.d.), range	39.59 (11.61), 18–69	31.46 (11.24), 18–61	29.83 (8.53), 18–60	30.29 (10.14), 19–63	Welch F (3, 113) = 11.79, *P* < 0.001, *η^2^* = 0.214	Autism > Autism + REDs, *P* < 0.001, *g* = 0.71 Autism > REDs, *P* < 0.001, *g* = 0.97 Autism > HATs + REDs, *P* < 0.001, *g* = 0.84
Ethnicity	Any white *n* (%)	58 (84.1%)	52 (91.2%)	77 (96.3%)	37 (97.4%)	Fisher–Freeman–Halton Exact Test (2-sided): *P* = 0.035	
	Other *n*(%)	11 (15.9%)	5 (8.8%)	3 (3.8%)	1 (2.6%)		
Highest level of education	No qualifications/GCSE	3 (4.3%)	7 (12.3%)	7 (8.8%)	4 (10.5%)	Pearson *χ²*(9) = 21.44, *P* = 0.011, *ϕc* = 0.171	
	A level or foundation degree	13 (18.8%)	26 (45.6%)	32 (40%)	16 (42.1%)		
	Bachelor's degree	30 (43.5%)	11 (19.3%)	28 (35%)	10 (26.3%)		
	Master's degree or PhD	23 (33.3%)	13 (22.8%)	13 (16.3%)	8 (21.1%)		
Type of REDs diagnosis	Anorexia nervosa	NA	*n* = 46/57, 80.7%;	*n* = 69/80, 86.3%	*n* = 33/38, 86.8%		
	Atypical anorexia nervosa	NA	*n* = 5/57, 8.8%	*n* = 11/80, 13.8%	*n* = 5/38, 13.2%		
	ARFID	NA	*n* = 6/57, 10.5%	*n* = 0/80. 0%	*n* = 0/38, 0%		
Age of diagnosis and REDs illness duration	Age of autism diagnosis mean (s.d.), range	35.03 (13.23), 8–68	27.96 (11.77), 11–58 (*n* = 55)	NA	NA	*t*(121) = 3.093, *P* = 0.002, *g* = 0.56, 95% CI [2.54–11.59]	
	Age of ED diagnosis mean (s.d.), range	NA	19.52 (7.87), 9–54 (*n* = 54)	22.61 (8.47), 11–54	20.97 (9.88), 11–59	*F*(2, 169) = 2.11, *P =* 0.125, *η*^2^ = 0.024	
	Age ED symptoms start mean (s.d.), range	NA	15.74 (7.83), 4–53 (*n* = 53)	17.03 (6.34), 7–44 (*n* = 79)	16.45 (7.97), 3–46	*F*(2, 167) = 0.51, *P =* 0.602, *η*^2^ = 0.040	
	Illness duration (years since ED diagnosis)^b^ mean (s.d.), range	NA	11.91 (11.93), 1–52 (*n* = 54)	7.21 (7.43), 0–29	9.32 (8.09), 1–33	Welch *F*(2, 86.07) = 3.57, *P* < 0.032, *η*^2^ = 0.116	Autism + REDs > REDs, *P* = 0.032, *g* = 0.49
	BMI^b^^,^^c^ mean (s.d.), range	27.91 (6.61), 15.24–43.41	18.12 (3.17), 13.11–30.04	17.26 (2.79), 12.34–26.20	17.28 (2.47), 11.76–23.26	Welch *F*(3, 105.87) = 49.79, *P* < 0.001, *η*^2^ = 0.550	Autism > Autism + REDs, *P <* 0.001, *g* = 1.82 Autism > REDs, *P <* 0.001, *g* = 2.17 Autism > HATs + REDs, *P <* 0.001, *g* = 1.91
Current mental health	HADS depression^b^^,^^d^ mean (s.d.), range	7.13 (4.29), 0–18	10.21 (5.56), 0–21	9.84 (3.92), 1–18	12.05 (3.98), 1–20	*F*(3, 239) = 11.917, *P* < 0.001, *η*^2^*_p_* = 0.130	Autism < Autism + REDs, *P* < 0.001, *g* = 0.63 Autism < REDs, *P* < 0.001, *g* = 0.66 Autism < HATs + REDs, *P* < 0.001, *g* = 1.18
	HADS anxiety^b^^,^^d^ mean (s.d.), range	11.52 (4.41), 3–21	14.95 (4.37), 2–21	13.85 (3.48), 5–20	15.63 (3.01), 9–21	*F*(3, 239) = 11.558, *P* < 0.001, *η*^2^*_p_* = 0.127	Autism < Autism + REDs, *P* < 0.001, *g* = 0.78 Autism < REDs, *P* = 0.003, *g* = 0.59 Autism < HATs + REDs, *P* < 0.001, *g* = 1.03
	SPIN^c^^,^^d^ mean (s.d.), range	37.23 (13.45), 0–64	45.60 (12.34), 6–68	36.53 (13.45), 5–65	46.66 (12.98), 6–68	*F*(3, 240) = 8.89, *P* < 0.001, *η*^2^*_p_* = 0.100	Autism < Autism + REDs, *P* = 0.016, *g* = 0.65 Autism < HATs + REDs, *P* = 0.016, *g* = 0.71 Autism + REDs > REDs, *P* < 0.001, *g* = 0.70 REDs < HATs + REDs, *P* < 0.001, *g* = 0.76

a. Tests of main effects used standard parametric tests, depending on whether there were 2 groups (T-test) or ≥3 groups (analysis of variance), unless otherwise stated.

b. Assumption of homogeneity of variance not met.

c. Reduced sample sizes due to missing BMI data: Autism (*n* = 64), Autism + REDs (*n* = 51), REDs (*n* = 77), HATs + REDs (*n* = 33).

d. Covariate is evaluated at the following value: Age (years) = 33.04.

ARFID, avoidant/restrictive food intake disorder; ED, eating disorder; REDs, restrictive eating disorders; GCSE, General Certificate of Secondary Education; HATs, high autistic traits; BMI, body mass index; HADS, Hospital Anxiety and Depression Scale; SPIN, Social Phobia Inventory.

### Correlation between autistic traits and BMI

Spearman's rank-order correlations showed no significant correlations between any autistic traits and BMI in each group (see Supplementary Appendix 5, sTable 4).

### Autistic characteristics

Table 2 shows group comparisons of mean total scores on measures of autistic characteristics (autistic traits in adulthood and childhood, restricted and repetitive behaviours, camouflaging behaviours), adjusting for differences in mean age between the groups. There was a significant effect of the group on each measure of autistic characteristics. For each measure, participants in the Autism + REDs and Autism groups scored higher than REDs participants. Both autistic groups showed similar levels of autistic characteristics, with one exception: Autism + REDs participants reported higher levels of restricted and repetitive behaviours than Autism participants. Overall, those with a RED who did not have an autism diagnosis but did have high autistic traits (HATs + REDs) showed a similar pattern of autistic characteristics, including childhood traits, to the diagnosed autistic participants. The only exception was on the adult autism spectrum quotient, where they scored significantly lower than autistic participants with and without REDs. HATs + REDs participants scored significantly higher than REDs participants on all autistic characteristics measures.

**Table 2:** Mean total scores, F statistic and post-hoc comparisons (adjusted for differences in age) for each autistic characteristic measure

Measure	Mean (s.d.)				Effect of group^a^	Significant post-hoc comparisons (Bonferroni correction applied for multiple comparisons with adjusted significance level at *P* ≤ 0.0083)
	Autism (*n* = 69)	Autism + REDs (*n* = 57)	REDs (*n* = 80)	HATs + REDs (*n* = 38)		
AQ^b^	38.15 (6.70)	38.49 (4.70)	20.91 (5.94)	32.97 (6.76)	*F*(3, 194) = 84.54, *P* < 0.001, *η*^2^*_p_* = 0.641	Autism > REDs, *P* < 0.001, *g* = 2.78 Autism > HATs + REDs, *P* = 0.010, *g* = 0.77 Autism + REDs > REDs, *P* < 0.001, *g* = 3.16 Autism + REDs > HATs + REDs, *P* < 0.001, *g* = 0.96 REDs < HATs + REDs, *P* < 0.001, *g* = 1.94
RAADS-14 childhood ratio	0.92 (0.15)	0.93 (0.13)	0.63 (0.36)	0.85 (0.19)	*F*(3, 239) = 23.05, *P* < 0.001, *η*^2^*_p_* = .224^c^	Autism > REDs *P* < 0.001, *g* = 1.02, 95% CI [0.18–0.40] Autism + REDs > REDs, *P* < 0.001, *g* = 1.04 REDs < HATs + REDs, *P* = 0.001, *g* = 0.70
RBQ-2A^c^	2.06 (0.41)	2.29 (0.32)	1.57 (0.31)	2.03 (0.37)	*F*(3, 239) = 50.75, *P* < 0.001, *η*^2^*_p_* = .389^c^	Autism < Autism + REDs, *P* = 0.006, *g* = 0.59 Autism > REDs, *P* < 0.001, *g* = 1.36 Autism + REDs > REDs, *P* < 0.001, *g* = 2.26 Autism + REDs > HATs + REDs, *P* = 0.002, *g =* 0.73 REDs < HATs + REDs, *P* < 0.001, *g* = 1.39
CAT-Q	124.26 (23.39)	130.35 (20.50)	96.60 (22.89)	120.50 (23.31)	*F*(3, 239) = 32.51, *P* < 0.001, *η*^2^*_p_* = 0.290	Autism > REDs, *P* < 0.001, *g* = 1.12 Autism + REDs > REDs, *P* < 0.001, *g* = 1.54 REDs < HATs + REDs, *P* < 0.001, *g* = 1.04

a. Covariate is evaluated at the following value: Age (years) = 33.04.

b. Reduced sample size for AQ comparison due to missing data: Autism (*n* = 40), Autism + REDs (*n* = 39), REDs (*n* = 79), HATs + REDs (*n* = 36).

c. Assumption of homogeneity of variance not met.

REDs, restrictive eating disorders; HATs, high autistic traits; AQ, Adult Autism Spectrum Quotient; RAADS-14, Ritvo Autism Asperger Diagnostic Scale; RBQ-2A, Adult Repetitive Behaviours Questionnaire; CAT-Q, Camouflaging Autistic Traits Questionnaire.

### Disordered eating symptoms

Age-adjusted group comparisons on measures of disordered eating symptoms (traditional eating disorder symptoms, body image issues, pride in eating pathology, autism-specific unusual eating behaviours) are presented in Table 3. There was a significant effect of the group on each measure indicating differences in traditional eating disorder symptoms, body image issues, pride in eating pathology and autism-specific unusual eating behaviours. The Autism + REDs group, compared with the Autism group, scored higher across all measures of disordered eating symptoms. In terms of traditional disordered eating symptoms, Autism + REDs participants scored similarly to the REDs group. However, although not significant, there was a consistent pattern of Autism + REDs participants presenting with lower levels of eating disorder symptoms compared to the REDs group, with small to medium effect sizes: traditional eating disorder symptoms (*P* = 0.095; *g* = 0.40; 95% CI [−0.05–1.13]), body image issues (*P* = 0.173; *g* = 0.38, 95% CI [−2.73–28.79]) and pride in their eating disorder (*P* = 0.66; *g* = 0.26; 95% CI [−4.57–18.45]). Notably, in the sensitivity analysis using only complete cases, the difference between Autism + REDs participants (mean = 3.51, s.d. = 1.43) and REDs participants (mean = 4.10, s.d. = 1.18) for traditional eating disorder symptoms was significant (*P* = 0.041, *g* = 0.46; 95% CI [0.016–1.192] with Bonferroni correction applied for multiple comparisons).

**Table 3:** Unadjusted mean total scores, F statistic and post-hoc comparisons (adjusted for differences in age) for disordered eating-related measure.

Measure	Mean (s.d.)				Effect of group^a^	Significant post-hoc comparisons (Bonferroni correction applied for multiple comparisons with adjusted significance level at *P* ≤ 0.0083)
	Autism (*n* = 69)	Autism + REDs (*n* = 57)	REDs (*n* = 80)	HATs + REDs (*n* = 38)		
EDE-Q global	1.78 (1.30)	3.56 (1.48)	4.09 (1.18)	4.49 (1.10)	*F*(3, 239) = 48.95, *P* < 0.001, *η*^2^*_p_* = 0.381	Autism < Autism + REDs, *P* < 0.001, *g* = 1.29 Autism < REDs only, *P* < 0.001, *g* = 1.87 Autism < HATs + REDs, *P* < 0.001, *g* = 2.20 Autism + REDs < HATs + REDs, *P* = 0.004, *g* = 0.69
BSQ	89.93 (31.62)	125.96 (35.33)	139.43 (35.98)	152.08 (32.76)	*F*(3, 239) = 29.37, *P* < 0.001, *η*^2^*_p_* = 0.269	Autism < Autism + REDs, *P* < 0.001, *g* = 1.08 Autism < REDs, < 0.001, *g* = 1.45 Autism < HATs + REDs, *P* < 0.001, *g* = 1.94 Autism + REDs < HATs + REDs, *P* = 0.002, *g* = 0.76
PEP-S	59.50 (21.89)	76.05 (27.89)	83.00 (25.83)	85.39 (23.01)	*F*(3, 239) = 11.92, *P* < 0.001, *η*^2^*_p_* = 0.130	Autism < Autism + REDs, *P* = 0.003, *g* = 0.67 Autism < REDs, *P* < 0.001, *g* = 0.98 Autism < HATs + REDs, *P* < 0.001, *g* = 1.16
SWEAA total	33.13 (12.93)	50.15 (11.71)	36.90 (12.70)	50.35 (10.24)	*F*(3, 239) = 30.15, *P* < 0.001, *η*^2^*_p_* = 0.275	Autism < Autism + REDs, *P* < 0.001, *g* = 1.37 Autism < HATs + REDs, *P* < 0.001, *g* = 1.42 Autism + REDs > REDs, *P* < 0.001, *g* = 1.08 REDs < HATs + REDs, *P* < 0.001, *g* = 1.12

a. Covariate is evaluated at the following value: Age (years) = 33.04.

REDs, restrictive eating disorders; HATs, high autistic traits; EDE-Q, Eating Disorder Examination-Questionnaire; BSQ, Body Shape Questionnaire; PEP-S, Pride in Eating Pathology Scale; SWEAA, SWedish Eating Assessment for Autism Spectrum Disorders.

Compared to the REDs and the Autism group, Autism + REDs participants had more autism-specific unusual eating behaviours, as measured by the SWEAA.

The HATs + REDs group attained the highest score on each disordered eating measure, indicating both severe traditional eating disorder symptoms and high levels of autism-specific unusual eating behaviours.

### SWEAA subscales

To better understand the nature of autism-specific risk factors for REDs, we looked at the pattern of group differences for each SWEAA subscale. The results of this analysis are shown in Supplementary Appendix 6 with group compositions being presented in Table 4. We were particularly interested to see on which subscales the Autism + REDs group scored significantly higher than both the Autism and the REDs group, given that such a pattern could give clues to autism-specific risk factors for REDs. This pattern was observed for the following SWEAA subscales: perception, purchase of food, eating behaviour, mealtime surroundings and social situations at mealtimes. By contrast, on the motor control and simultaneous capacity subscales, the Autism and the Autism + REDs group were indistinguishable, but both scored significantly higher than the REDs group; and on the disturbed eating behaviour subscale, Autism + REDs participants scored similarly to REDs participants, with the Autism group scoring significantly lower. Overall, the Autism + REDs and the HATs + REDs group presented similarly across SWEAA subscales.

**Table 4:** Unadjusted mean SWEAA subscale scores and post-hoc comparisons for the model adjusted for differences in age

Measure	Mean (s.d.)				Significant pairwise comparisons (Bonferroni correction applied for multiple comparisons with adjusted significance level at *P* ≤ 0.0083)
	Autism (*n* = 69)	Autism + REDs (*n* = 57)	REDs (*n* = 80)	HATs + REDs (*n* = 38)	
SWEAA perception	47.33 (21.55)	59.29 (19.21)	36.65 (19.11)	57.36 (16.61)	Autism < Autism + REDs, *P* = 0.004, *g* = 0.58 Autism > REDs, *P* = 0.021, *g* = 0.53 Autism + REDs > REDs, *P* < 0.001, *g* = 1.18 REDs < HATs + REDs, *P* < 0.001, *g* = 1.13
SWEAA motor control^a^	22.83 (17.23)	26.63 (19.35)	12.95 (11.82)	23.33 (15.33)	Autism > REDs, *p* < 0.001, *g* = 0.68 Autism + REDs > REDs, *P* < 0.001, *g* = 0.89 REDs < HATs + REDs, *P* = 0.006, *g* = 0.80
SWEAA purchase of food^a^	42.03 (25.78)	70.76 (23.52)	48.75 (29.02)	66.67 (20.96)	Autism < Autism + REDs, *p* < 0.001, *g* = 1.16 Autism < HATs + REDs, *P* = 0.001, *g* = *1.02* Autism + REDs > REDs, *P* < 0.001, *g* = 0.82 HATs + REDs > REDs, *P* = 0.003, *g* = 0.67
SWEAA eating behaviour	35.57 (20.86)	62.72 (18.66)	49.84 (21.68)	64.04 (19.04)	Autism < Autism + REDs, *P* < 0.001, *g* = 1.37 Autism < REDs, *P* < 0.001, *g* = 0.69 Autism < HATs + REDs, *P* < 0.001, *g* = 1.41 Autism + REDs > REDs, *P* = 0.003, *g* = 0.63 REDs < HATs + REDs, *P* = 0.003, *g* = 0.68
SWEAA mealtime surroundings	35.64 (20.63)	63.36 (16.89)	48.32 (22.18)	63.70 (18.17)	Autism < Autism + REDs, *P* < 0.001, *g* = 1.46 Autism < REDs, *P* = 0.001, *g* = 0.59 Autism < HATs + REDs, *P* < 0.001, *g* = 1.42 Autism + REDs > REDs, *P* < 0.001, *g* = 0.75 REDs < HATs + REDs, *P* < 0.001, *g* = 0.73
SWEAA social situations at mealtime	37.32 (13.13)	49.08 (14.58)	39.13 (13.31)	48.82 (13.32)	Autism < Autism + REDs, *P* < 0.001, *g* = 0.85 Autism < HATs + REDs, *P* < 0.001, *g* = 0.87 Autism + REDs > REDs only, *P* < 0.001, *g* = 0.72 REDs < HATs + REDs, *P* = 0.002, *g* = 0.73
SWEAA other disturbed eating behaviours^a^	11.21 (10.99)	32.18 (12.91)	30.00 (12.78)	39.80 (19.45)	Autism < Autism + REDs, *P* < 0.001, *g* = 1.76 Autism < REDS only, *P* < 0.001, *g* = 1.57 Autism < HATs + REDs, *P* < 0.001, *g* = 1.96 Autism + REDs < HATs + REDs, *P* = 0.048, *g* = 0.48 REDs < HATs + REDs, *P* = 0.002, *g* = 0.50
SWEAA hunger and satiety	36.41 (24.61)	46.49 (24.18)	43.91 (22.32)	43.75 (21.70)	No significant differences
SWEAA simultaneous capacity	27.90 (30.48)	38.16 (30.66)	21.87 (28.51)	35.52 (30.55)	Autism + REDs > REDs, *P* = 0.014, *g* = 0.55

a. Assumption of homogeneity of variance not met.

REDs, restrictive eating disorders; HATs, high autistic traits; SWEAA, SWedish Eating Assessment for Autism Spectrum Disorders.

## Discussion

The current study is the first to quantitatively describe the clinical presentation of autistic individuals with REDs compared with autistic individuals without REDs and non-autistic women with REDs. We aimed to increase understanding of what underpins REDs in autistic women and to support recognition of these individuals in eating disorder services. In addition, given issues of diagnostic overshadowing, a group of women with REDs who had no autism diagnosis but did report high autistic traits was included to explore their similarities to individuals with REDs and an autism diagnosis.

There were several similarities between autistic and non-autistic individuals with REDs in terms of BMI, levels of general anxiety, depression and some disordered eating symptoms. An important difference, however, was that the former scored substantially higher on a measure of autism-specific eating behaviours (i.e. the SWEAA). Crucially, the Autism + REDs group also had higher levels of these behaviours compared with the Autism group. This suggests that such autism-specific eating difficulties are not simply a general feature of autism but, rather, are characteristic of that subset of autistic women who have developed clinical REDs. In particular, autistic individuals with REDs presented with higher scores on SWEAA subscales related to sensory-driven food restriction; insistence on sameness and intolerance of uncertainty; inflexible, ritualistic behaviour at mealtimes; as well as the environment and social interactions during mealtimes. Similarly, autistic individuals with REDs reported higher levels of social anxiety than both autistic individuals without REDs and non-autistic women with REDs. In addition, of the autistic characteristics measured in the current study, restricted and repetitive behaviours were the only characteristics endorsed more strongly by autistic participants with REDs than those without REDs, thus suggesting that these could be implicated in REDs development and/or maintenance. This profile fits with qualitative findings based on the lived experience of autistic women with REDs.^13,36^ It is also consistent with a previous study on the nature of autistic traits in young people with anorexia nervosa;^37^ and the observation that a range of conditions associated with rigid behaviour and unusual sensory processing are associated with REDs.^38^

It is noteworthy that autistic individuals with REDs also presented with high levels of camouflaging behaviour (mean = 130.34), although there was no significant difference from the group of autistic women without REDs (mean = 124.26), with this group also scoring high compared with other autistic women from community samples (e.g. mean = 114 in^39^). Nonetheless, future research should consider the role of camouflaging and associated distress,^40,41^ as well as other autism-related factors not explored in this study (e.g. differences social relating and executive functioning) for REDs in autistic women.

This is, to our knowledge, the first study to test the idea, suggested by qualitative research,^13^ that autistic women with REDs have fewer weight and shape concerns compared with non-autistic women with REDs. We found that the Autism + REDs group showed a pattern of lower scores on measures of traditional eating disorder symptoms related to weight and shape concerns, body image issues and pride in eating pathology, although this did not reach significance. Further, compared with autistic women without REDs, Autism + REDs participants did score significantly higher on these measures. Therefore, based on the current findings, there is evidence that weight and shape concerns have a role in the onset and/or maintenance of REDs for at least some autistic women. Future research should explore this further, including whether weight and shape concern-related eating disorder symptoms in autistic women with REDs might be qualitatively different, e.g. their onset might be secondary to more typical autistic eating difficulties, or whether there is a subgroup without overt weight and shape concerns.^42^

This study offers insights into the identification and understanding of autistic women in eating disorder settings. As discussed above, the presence of unusual eating behaviours in addition to more traditional eating disorder symptoms appears to be a good indicator that someone might be autistic. Another obvious difference between autistic and non-autistic individuals with REDs is that the autistic participants scored much higher across measures of autistic characteristics. Yet, camouflaging behaviour might make them seem less autistic, but could be an additional source of stress and exhaustion for these individuals.^40^

Concerns have been raised that having a RED might cause non-autistic women to erroneously score highly on autistic trait measures.^43^ However, the fact that women with REDs identified through scores above a conservative cut-off on an autism screening measure (HATs + REDs) presented similarly to individuals with a formal autism diagnosis in terms of other clinical characteristics, suggests that even brief self-report measures can measure autistic traits as distinct from RED and other clinical characteristics in eating disorder populations. This is further supported by the lack of correlation between BMI and autistic traits, which is in line with findings from other research,^44^ as a significant negative correlation would have been consistent with the idea that these are owing to starvation rather than autism. In addition, the fact that higher levels of autistic traits in this group have been present in childhood suggests that they likely pre-dated the onset of the eating disorder, although the limitation of self- rather than informant-reported childhood traits should be noted here.

Among participants initially recruited as women with REDs (*n* = 118) without a formal autism diagnosis, around one-third (*n* = 38) presented with very high autistic traits (HATs + REDs). Although online recruitment might have induced some bias, this is in line with prevalence estimates from other studies with more representative samples^3^ and qualitative accounts of delay in autism diagnosis in women with REDs.^11^ As a group, these women showed high levels of: autistic traits, including in childhood, and associated characteristics (i.e. restricted and repetitive behaviours, camouflaging behaviours); distress, as indexed by their high levels of general anxiety, depression and social anxiety; traditional eating disorder symptoms; and autism-specific eating difficulties. In short, they looked very similar to participants with a RED and a formal autism diagnosis. The only exception was that they had lower scores on one of the measures of general autistic traits (adult autism spectrum quotient), although their mean score (mean = 32.97) was still above that measure's screening threshold of 26.^25^ A plausible explanation for this is that many HATs + REDs participants might be undiagnosed autistic women. This would need to be confirmed with a full diagnostic assessment, including gold-standard measures and a developmental history. It was a limitation of the current study that, because of the COVID-19 pandemic, we had to rely on self-report measures and were unable include in-person observational measures. This would have allowed us to confirm self-reported autism diagnostic status across the sample and would have provided further evidence as to whether participants in the HATs + REDs group are likely to be undiagnosed autistic women. While online recruitment allowed for the largest sample in this area of research thus far, it might have affected representativeness of the sample. It should also be noted that, as unfortunately is often the case in autism and eating disorder research,^45,46^ the current sample is predominantly white and highly educated, thus implicating transferability of results to other patient groups.

### Clinical implications

First, our findings are supportive of the use of a portfolio of self-report autism measures (including those assessing camouflaging and autism-specific unusual eating behaviours) to inform decisions about whether a client with RED might be autistic and would benefit from treatment adaptations. Conducting formal autism diagnostic assessments in currently unwell individuals with REDs is challenging, and this challenge is currently compounded by long waiting times in autism diagnostic services.^47^ Therefore, it might be sufficient to rely on the recognition of autistic traits to inform treatment adaptations,^48^ with the option to conduct a full diagnostic autism assessment at later stages. This supports recent thinking in the autism field, which favours a more dimensional, heterogeneous characterisation of autistic individuals over a rigid categorical approach,^49^ and could make support more accessible for a greater proportion of individuals.

Second, autism-related disordered eating symptoms should be routinely considered for assessment and treatment (see ^50,51^ for examples and guidance). When assessing autistic women's RED presentations, a narrow focus on traditional disordered eating symptoms might overlook or underestimate additional significant eating issues. If treatment only targets more traditional disordered eating symptoms, the persistence of other autism-specific eating behaviours might hinder progress towards recovery or increase risk of relapse.

Third, we present the strongest evidence to date for putative autism-specific risk factors for REDs, related to autistic sensory, flexibility and social differences. Longitudinal research is required to test whether these play a causal role in the development of REDs of autistic women.

## Supporting information

Brede et al. supplementary material Brede et al. supplementary material

## Data Availability

The corresponding author will consider requests from researchers for access to the fully anonymised data-set used in this study.
