# Supplementary material for: The clinical characteristics of autistic women with restrictive eating disorders
Source: BJPsych Open. 2024 Jul 26;10(4):e131. doi: 10.1192/bjo.2024.65 (PMC11698150; doi:10.1192/bjo.2024.65)
Supplement: Brede et al. supplementary material [file S2056472424000656sup001.docx]

Appendix and supplementary tables

Appendix 1

*Measures taken to minimise impact of COVID-19*

The COVID-19 pandemic and related lockdown and social distancing restrictions arrived in the UK after we had started data collection. We reviewed how COVID-19 might affect the applicability of questionnaire items and participants experience of the constructs being measured. For example, social distancing measures were likely to affected participant’s ability to and experience of socialising, which could affect responses to certain measures of autistic characteristics. To minimise biased responses, specific guidelines were added to questionnaire items likely to be affected by COVID-19. These acknowledged the potential impact of COVID-19 on the construct being measured and asked participants to ‘respond based on your experience in the recent past prior to the current COVID-19 situation’.

Our initial data collection protocol included the autism diagnostic observational scale (ADOS-2, (1)), a gold standard autism assessment, in addition to self-report measures completed via the online survey. This would have provided an autism threshold score to support the autism diagnostic status of participants with a formal autism diagnosis, the lack of an autism diagnosis among participants allocated to the REDs group, and to identify women with REDs and no autism diagnosis who should be assigned to the HATs+REDs group. However, COVID-19 meant we were no longer able to collect data in-person. Instead, we used the RAADS-14 (2) for group allocation and added another more extensive self-report measure of autistic traits, namely the AQ (3). This is only available for participants recruited after the onset of COVID-19.

There were some minor differences on demographic or clinical variables in each group for those participating before or during COVID-19. However, these did not show a consistent pattern and did not survive correction for multiple comparison.

Appendix 2

**sTable 1**

*Details on The SWedish Eating Assessment for Autism Spectrum Disorders (SWEAA; (35)) subscales*

| **SWEAA subscale** | **Items** | **Description** |
| --- | --- | --- |
| A Perception | 11 | Includes items on sensitivity to sensory experiences of eating (e.g. taste, texture of food, sound of chewing, mixing of different ingredients, and visual presentation on plate). |
| B Motor control | 7 | Includes items on ability to chew, hand-eye coordination, and spilling off food/drink. |
| C Purchase of food | 3 | Includes items on desire for control over food purchased and preference for certain supermarket chains or brands |
| D Eating behaviour | 6 | Includes items on eating routines, food neophobia, and restriction of variety of food items/dishes. |
| E Mealtime surroundings | 11 | Includes items on preference for certain utensils or seating arrangements, rituals around mealtime, eating in the company of others and in different environments (i.e. outside the home). |
| G Social situation at mealtime | 10 | Includes items on social interactions during mealtime and adherence to socially accepted table manners. |
| H Other behaviour associated with disturbed eating | 8 | Includes items on disordered eating behaviours, such as vomiting, restriction, and food refusal or substitution. |
| I Hunger/ satiety | 2 | Includes items on interoeptive sensivity. |
| J Simultaneous capacity | 1 | Asks about ability to do two things simultaneously during meal time (e.g. chewing and cutting the food). |
| K Pica | 1 | Asks about eating of things others consider inedible (e.g. soil). |

Appendix 3

*Inclusion criteria for each participant group and verification of diagnostic status*

Inclusion criteria for each participant group are listed in sTable 1.

**sTable 2**

*Inclusion criteria for each participant group.*

| Criteria | Autism | Autism+REDs | REDs/ HATs+REDs |
| --- | --- | --- | --- |
| Gender | Female, including non-binary and trans-female gender identities | Female, including non-binary and trans-female gender identities | Female, including non-binary and trans-female gender identities |
| Age | 18+ | 18+ | 18+ |
| Intellectual ability | No Intellectual disability (ID) | No ID | No ID |
| Autism diagnostic status | Must have a clinical autism diagnosis (including autism spectrum disorder, autism, Asperger’s syndrome, high functioning autism, and pervasive developmental disorder) | Must have a clinical autism diagnosis (including autism spectrum disorder, autism, Asperger’s syndrome, high functioning autism, and pervasive developmental disorder) | No autism diagnosis |
| RED diagnostic status | No (past or current) RED diagnosis | Must be clinically diagnosed and currently living with a RED (including anorexia nervosa, atypical anorexia and ARFID) and score above threshold on study ED questionnaires | Must be clinically diagnosed and currently living with a RED (including anorexia nervosa, atypical anorexia and ARFID) ) and score above threshold on study ED questionnaires |

Participants with REDs confirmed during screening that they were clinically diagnosed and currently living with a RED (including AN, atypical anorexia and ARFID). We used secondary criteria, applied to the collected data, to confirm the presence of current ED symptoms. Participants with REDs were required to score above a clinically meaningful cut-off on at least one of three pre-selected disordered eating-related (sub)scales. We considered scores on a combination of measures, which focus on different mechanisms underlying disordered eating, to prevent exclusion of participants with less traditional RED presentations, that is, those with fewer weight and shape concerns. Participants must have scored above the widely used cut-off of 2.5 on the EDE-Q global score (4, 5) and/or +1 standard deviation above the means of an autistic (clinical) group, derived from normative data in the original validation study (6), on the SWEAA eating behaviour subscale or the SWEAA other behaviour associated with disturbed eating subscale (6). In addition, participants reporting an AN diagnosis had to be either currently underweight, i.e. BMI below 18.5, or to have been underweight at their lowest ever weight, in line with DSM-5 criteria.

Autistic participants confirmed during screening that they had an independent formal autism diagnosis. Diagnoses had to be given by a qualified health care professional or multi-disciplinary team in line with latest ICD or DSM criteria at the time of their assessment. For participants recruited from clinical settings, medical records were reviewed by the medical team supporting recruitment to confirm autism diagnosis. In line with other influential studies (7-9), we asked participants recruited via charities or social media to disclose additional information (e.g. type of practitioner who diagnosed them, year of their diagnosis, their specific diagnosis) in order to verify their diagnosis. Mean scores on autistic trait measures were supportive of genuine diagnoses in the autism groups (see Table 2).

Appendix 4

*Sensitivity analysis*

We conducted post-hoc sensitivity analyses to determine the minimum effect size each group comparison was powered to detect based on the final acquired sample sizes with power level of 80% and two-tailed alpha at .05. This was conducted using G*Power version 3.1.9.2 (Faul et al., 2007)*.* As shown in sTable 2, group comparisons in the final sample were powered to detect differences of a medium effect size.

sTable 3

*Effect size needed for each group comparison to be sufficiently powered based on the final acquired sample size*

| **Group comparison** | **Minimum effect size required (*d*)** |
| --- | --- |
| Autism vs Autism+REDs | 0.51 |
| Autism vs REDs | 0.46 |
| Autism vs HATs+REDs | 0.57 |
| Autism+REDs vs REDs | 0.49 |
| Autism+REDs vs HATs+REDs | 0.59 |
| REDs vs HATs+REDs | 0.56 |

*Note.* Autism (n=69), Autism+REDs (n=57), REDs (n=80), HATs+REDs (n=38)

REDs= Restrictive eating disorders; HATs=high autistic traits

Appendix 5

sTable 4

*Correlations between total RAADS-14 scores and BMI for each group*

|  | **BMI** |
| --- | --- |
| Autism only (n = 64) ^‡^ | *r_s_* = .223, [-.031-.458], *p* = .077 |
| Autism+REDs (n = 51) ^‡^ | *r_s_* = .004, [-.266-.277], *p* = .976 |
| REDs only (n = 77) ^‡^ | *r_s_* = .020, [-.217-.265], *p* = .860 |
| REDs high autistic traits (n = 33) ^‡^ | *r_s_* = -.054, [-.359-275], *p* = .765 |

*Note.*

The correlation coefficient (*r_s_)* for each correlation is reported as an indicator of strength of the bivariate relationship. Bootstrapped 95% CIs are reported in square brackets.

^‡^reduced sample sizes due to missing BMI data.

REDs= Restrictive eating disorders; HATs=high autistic traits; BMI=Body Mass Index

Appendix 6

*Main effects of subscale, group, and interaction for SWEAA subscale analysis*

Box’s test of equality of covariance was violated (Box’s *M* = 180.64, *F*(135, 74880.42) = 1.24, *p* = *.*030). According to Levene’s test, the assumption of quality of variance for individual subscale scores was met for all subscales, apart from SWEAA motor control and SWEAA disturbed eating behaviour. Mauchly’s test indicated that the assumption of sphericity had been violated (*χ*^2^(35) = 375.85, *p* < *.*001); therefore, Greenhouse-Geisser corrections were applied (*ε* = .69).

Main effects for subscale, group and interaction of subscale by group, adjusted for age, for SWEAA subscales are presented in sTable 4.

**sTable 5**

*Mixed-design ANOVA main effects of subscale, group, and interaction for SWEAA subscales adjusted for differences in age*

| **Mein effect** ^a^ | **Statistical Result** |
| --- | --- |
| Subscale | *F*(5.55, 1327.22) = 8.54, ***p* < *.*001**, *η*^2^*_p_* = .035 |
| Group | *F*(3, 239) = 26.32, ***p* < *.*001**, *η*^2^*_p_* = .248 |
| Subscale by group | *F*(16.66, 1327.22) = 7.27, ***p* < *.*001**, *η*^2^*_p_* = .084 |

^a^ Covariate is evaluated at the following value: Age (years) = 33.04

References

1. Lord C, Rutter M, Dilavore PC, Risi S, Gotham K, Bishop SL, et al. ADOS-2: Autism diagnostic observation schedule: Hogrefe.; 2008.

2. Eriksson JM, Andersen LM, Bejerot S. RAADS-14 Screen: validity of a screening tool for autism spectrum disorder in an adult psychiatric population. Mol Autism. 2013;4(1):49.

3. Baron-Cohen S, Wheelwright S, Skinner R, Martin J, Clubley E. The autism-spectrum quotient (AQ): evidence from Asperger syndrome/high-functioning autism, males and females, scientists and mathematicians. Journal of autism and developmental disorders. 2001;31(1):5-17.

4. Rø Ø, Reas DL, Stedal K. Eating Disorder Examination Questionnaire (EDE-Q) in Norwegian Adults: Discrimination between Female Controls and Eating Disorder Patients. Eur Eat Disord Rev. 2015;23(5):408-12.

5. Mond JM, Hay PJ, Rodgers B, Owen C, Beumont PJ. Validity of the Eating Disorder Examination Questionnaire (EDE-Q) in screening for eating disorders in community samples. Behav Res Ther. 2004;42(5):551-67.

6. Karlsson L, Rastam M, Wentz E. The SWedish Eating Assessment for Autism spectrum disorders (SWEAA)-Validation of a self-report questionnaire targeting eating disturbances within the autism spectrum. Res Dev Disabil. 2013;34(7):2224-33.

7. Crane L, Adams F, Harper G, Welch J, Pellicano E. 'Something needs to change': Mental health experiences of young autistic adults in England. Autism. 2019;23(2):477-93.

8. Hull L, Levy L, Lai M-C, Petrides KV, Baron-Cohen S, Allison C, et al. Is social camouflaging associated with anxiety and depression in autistic adults? Molecular Autism. 2021;12(1):13.

9. Weir E, Allison C, Baron-Cohen S. The sexual health, orientation, and activity of autistic adolescents and adults. Autism Research. 2021;14(11):2342-54.
